# Evaluation of Auranofin Loading within Ferritin Nanocages

**DOI:** 10.3390/ijms232214162

**Published:** 2022-11-16

**Authors:** Rosanna Lucignano, Alessandro Pratesi, Paola Imbimbo, Daria Maria Monti, Delia Picone, Luigi Messori, Giarita Ferraro, Antonello Merlino

**Affiliations:** 1 Department of Chemical Sciences, University of Naples Federico II, Complesso Universitario di Monte Sant’Angelo, Via Cinthia 21, 80126 Naples, Italy; 2 Department of Chemistry and Industrial Chemistry, University of Pisa, Via Giuseppe Moruzzi, 13, 56124 Pisa, Italy; 3 Department of Chemistry “Ugo Schiff”, University of Florence, Via della Lastruccia 3-13, Sesto Fiorentino, 50019 Florence, Italy

**Keywords:** gold compounds, metal complex, metallodrugs, protein metalation, ferritin encapsulation, anticancer activity

## Abstract

Auranofin (AF), a gold(I) compound that is currently used for the treatment of rheumatoid arthritis and is in clinical trials for its promising anticancer activity, was encapsulated within the human H-chain and the horse spleen ferritin nanocages using the alkaline disassembly/reassembly protocol. The aim of the work was to highlight possible differences in their drug loading capacity and efficacy. The drug-loaded ferritins were characterized via UV-vis absorption spectroscopy and inductively coupled plasma-atomic emission spectroscopy to assess AF encapsulation and to define the exact amount of gold atoms trapped in the Ft cavity. The crystal structures allowed us to define the nature of AF interaction with both ferritins and to identify the gold binding sites. Moreover, the biological characterization let us to obtain preliminary information on the cytotoxic effect of AF when bound to the human H-chain.

## 1. Introduction

**Figure 1:**
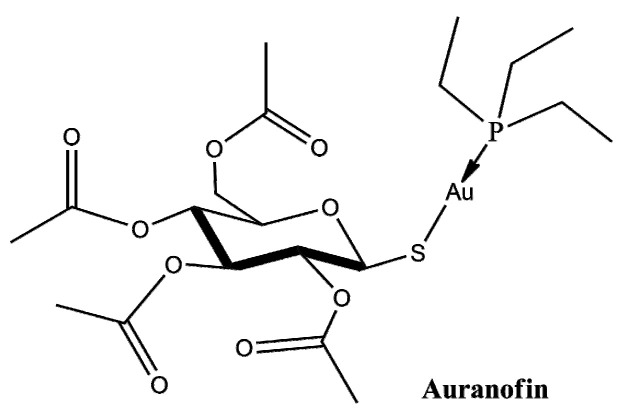
Schematic representation of Auranofin.

Auranofin (AF) is an FDA-approved drug for the treatment of rheumatoid arthritis [1] that has shown potent anticancer activity against many cancer cell lines [2,3,4]. Currently, it is being evaluated in phase I/II clinical trials for ovarian cancer [4], lung cancer [5] and leukemia [6]; its efficacy against the SARS-CoV-2 disease is also under consideration [7,8]. The drug complex consists of two parts: a water-soluble aurothioglucose entity with a sulfur donor group and a phosphine ligand that provides lipophilicity (Figure 1).

Due to its chemical nature, AF shows high selectivity for sulfur and selenium atoms, and thus for proteins with exposed free cysteines and selenocysteines [9,10]. AF acts as a pro-oxidant agent, disrupting the redox system of the cell by strongly inhibiting thioredoxin reductases (TrxRs) [11]. These enzymes act as antioxidants, regulating the levels of reactive oxygen species (ROS), thus protecting cells from the consequences of oxidative stress [1,12]. Interestingly, overexpression of TrxRs has been detected in several tumors [13,14] and is closely associated with tumorigenesis and a poor prognosis of the disease. Consequently, AF, targeting TrxRs, might be an interesting anticancer agent, promoting cancer cell death by altering the protective redox systems by which cancer cells can keep under control their abnormally high ROS levels. The anticancer activity of this drug could be further improved by using a carrier to deliver it to the final target. The targeted delivery of known chemotherapeutics to the tumor site has proved to be a promising strategy, even though it is in an early stage of clinical development. A smart drug delivery system allows the introduction of active drugs into the body in order to improve their efficacy as well as safety by controlling the dosage, duration of effect, release at the site of action, and successful crossing of biological barriers to reach the specific target [15]. Polymers, inorganic particles, lipids, and proteins that assemble to form supramolecular structures can be used for this scope. Among them, protein nanocages, such as ferritins (Fts), are particularly attractive [16]. Mammalian Fts are composed of two different chains, the H-chain (heavy) and the L-chain (light), that assemble to form a hollow cage-like structure with an internal diameter of 8 nm and an external one of 12 nm. This architecture is suitable for loading small molecules inside the internal cavity, taking advantage of several features. This carrier is biocompatible and non-immunogenic because it is already present in our body and is stable and soluble in the bloodstream. Furthermore, its smaller size when compared to other drug carriers could lead to a longer circulation half-life and better tumor accumulation rates [16]. Moreover, Ft possesses site-specific targeting potential, since it can be recognized and internalized by Ft-binding receptors such as the transferrin receptor 1 (TfR1) [17] and Scara5 [18], which are over-expressed in a variety of malignant cells [17]. Different strategies have been developed for the loading of drugs within ferritin cages. One of these methods takes advantage of ferritin stability in extreme pH conditions [19]. In this paper, the alkaline disassembly/reassembly protocol, already used to trap different metallodrugs within the Ft cage [20,21,22,23], has been used to produce AF-Ft nanocomposites. In particular, two different ferritins were selected for this kind of study: the human H-chain ferritin (hHFt) and the horse spleen ferritin (hsFt), which is mainly L-chain.

## 2. Results

### 2.1. Preparation and Characterization of Auranofin-Encapsulated Ferritins

The AF-encapsulated human H-chain Ft (_AF_hHFt) and horse spleen Ft (_AF_hsFt) were prepared using the alkaline disassembly/reassembly protocol as described in the Materials and Methods section and characterized by circular dichroism (CD), UV-vis absorption spectroscopy and inductively coupled plasma-atomic emission spectroscopy (ICP-AES).

**Figure 2:**
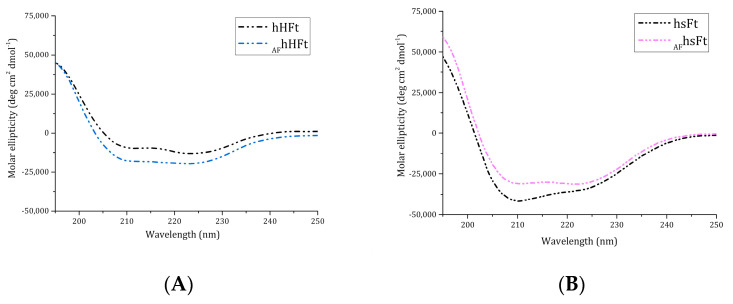
Far UV-CD spectra of _AF_hHFt ((**A**), light blue dashes) and _AF_hsFt ((**B**), pink dashes) in comparison to the spectra of the respective gold-free proteins (black dashes).

Far-UV CD spectra of _AF_hHFt and _AF_hsFt were collected to analyze the protein folding after the encapsulation procedure (Figure 2A,B). The superposition of the CD spectra of gold-loaded Fts with those of AF-free proteins showed that the experimental conditions used for AF encapsulation did not significantly affect the secondary structure content of Fts, since the proteins kept their fold upon drug encapsulation. 

**Figure 3:**
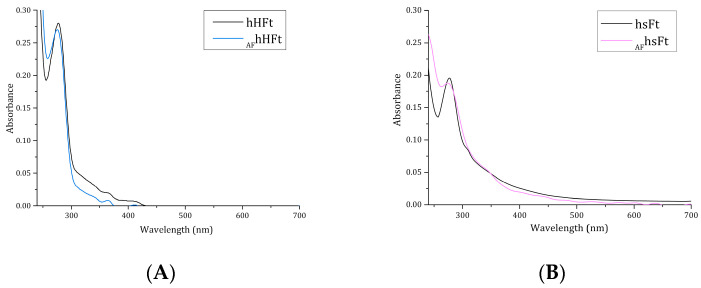
UV–vis spectra of _AF_hHFt ((**A**), light blue line) and _AF_hsFt ((**B**), pink line) in comparison to the spectra of the respective gold-free proteins (black lines).

It has been reported that an absorption increase in the region between 250 and 280 nm in the UV-vis spectrum of Fts indicates successful drug encapsulation [23]. For this reason, the UV-vis spectra of the AF-loaded nanocomposites were registered and compared with those of the respective drug-free Fts (Figure 3A,B). Different results were obtained for the two preparations. _AF_hsFt showed a significant absorbance increase between 250 and 280 nm when compared to the AF-free protein (Figure 3B), while only a slight variance of the absorbance occurred when the spectrum of _AF_hHFt was compared with that of hHFt (Figure 3A). This difference may be associated with a variation in the drug loading ability of the two Fts.

To explain the difference in the UV-vis spectra and to define with high accuracy the amount of AF that is encapsulated within the Ft nanocages, ICP-AES measurements were carried out. ICP-AES data indicated that about 270 gold atoms were encapsulated within _AF_hsFt, achieving a protein chain to metallodrug molar ratio of ~1:11, while only about 90 atoms of gold were found in the nanocage based on the human H-chain Ft, with a protein chain to metallodrug molar ratio of ~1:4. Therefore, the ICP-AES analysis is in line with the conclusions suggested by UV-vis absorption spectroscopy.

### 2.2. X-ray Structures of _AF_hHFt and _AF_hsFt

X-ray diffraction data were collected at 1.24 and 1.17 Å resolution on crystals of _AF_hsFt and _AF_hHFt, respectively. The crystals belong to the space group F432, with one Ft chain in the asymmetric unit. The structures refine to R_factor_ and R_free_ values within the ranges of 0.160–0.180 and 0.150–0.166 (Table 1). The overall conformation of both proteins in the crystals was not significantly affected by the presence of AF. The Cα root mean square deviations from the AF-free protein structures (PDB code 5ERK [20] for hsFt and 5N27 [24] for hHFt) were within the range 0.12–0.52 Å.

Positions of gold atoms in both structures were unambiguously identified by inspection of anomalous difference and Fourier difference (2Fo-Fc and Fo-Fc) electron density (e.d.) maps.

**Figure 4:**
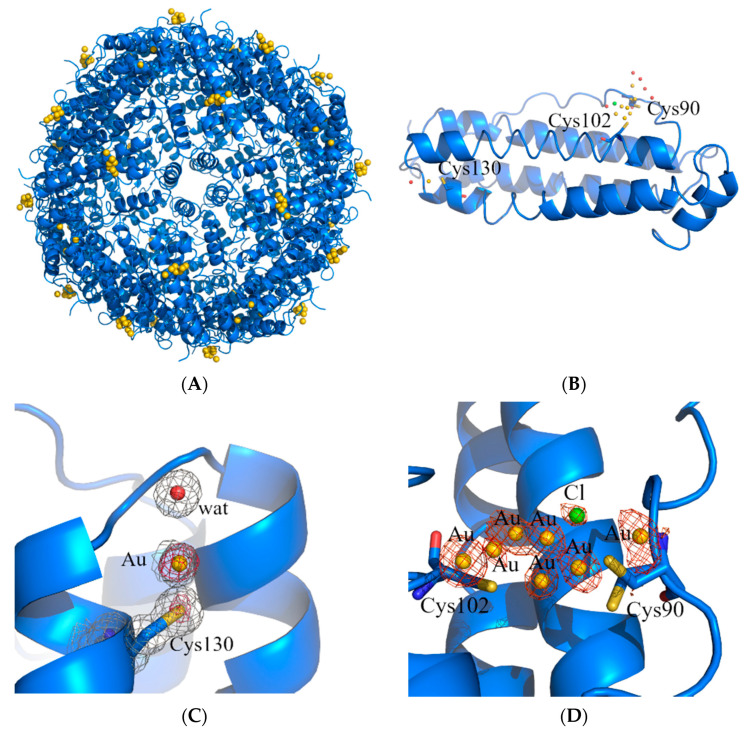
Cartoon representation of the structure of the cage (**A**) and of the single protein chain (**B**) of _AF_hHFt. The gold binding sites are shown in panels (**C**,**D**), and 2Fo–Fc and anomalous electron density maps are contoured at 1.0 σ (grey) and 3.0 σ (red), respectively.

In the structure of _AF_hHFt, two gold binding sites were found. The first one was close to Cys130. Here, a gold atom with low occupancy (0.15) was bound to the SG atom of the Cys and to a water molecule, showing a linear geometry (Figure 4C). The second binding site was on the outer surface of the cage, close to the side chains of Cys90 and Cys102 (Figure 4B,D). Here, several peaks in the anomalous difference e.d. map were found, suggesting the possibility that a “cluster” of gold atoms could be formed.

The structure of the potential gold cluster could not be exactly defined since the interpretation of the e.d. maps at this site is very hard, also because of the disordering of residues 89–94 in that region (Cys90 and Glu94 have been modeled in two alternative conformations). In our opinion, the best interpretation of the e.d. map is that reported in Figure 4D: there are four gold centers with partial occupancy very close to each other, two of which adopt a total of four alternative positions. In the same region, a peak of the anomalous difference e.d. map lower than those assigned to gold atoms was interpreted as a chloride ion with 0.20 occupancy (green sphere in Figure 3D). Although we cannot exclude that other interpretations of the map are possible, it is clear from the inspection of the 2Fo-Fc and anomalous difference e.d. maps that two gold atoms were directly coordinated to the side chains of Cys90 and Cys102. Considering that ICP-AES data indicated that _AF_hHFt contains about four gold atoms per hHFt chain and that at least three Cys residues (Cys90, Cys102 and Cys130) bind gold centers in the X-ray structure of _AF_hHFt, it can be concluded that only a small fraction of AF was encapsulated in the bulk of the hHFt nanocage.

**Figure 5:**
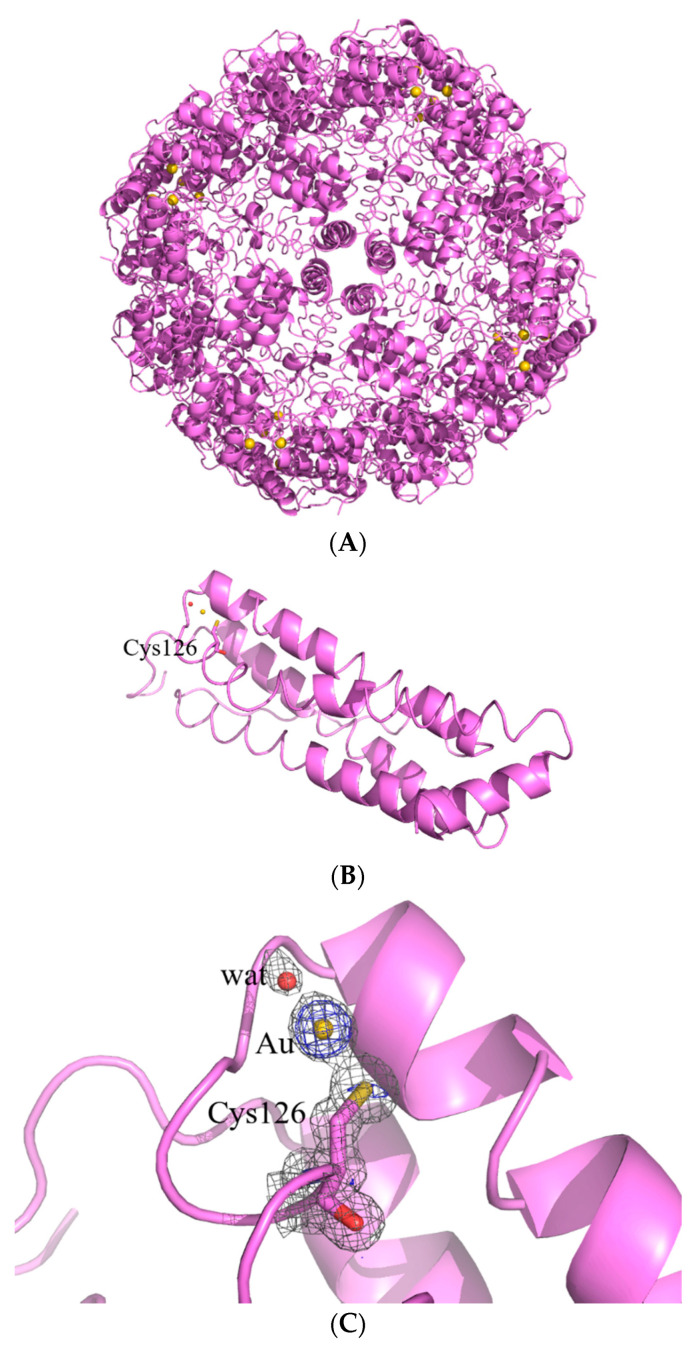
Cartoon representation of the structure of the cage (**A**) and of the single protein chain (**B**) of _AF_hsFt. The gold binding site is shown in panel (**C**), and 2Fo–Fc and anomalous electron density maps are contoured at 1.0 σ (grey) and 3.0 σ (blue).

At variance with what was observed for _AF_hHFt, the structure of _AF_hsFt presented a single gold binding site. A gold atom with 0.20 occupancy binds the SG atom of Cys126; a water molecule completes the metal coordination sphere, giving rise to a linear geometry (Figure 5B,C). In the case of _AF_hsFt, the comparison between the structural data and the ICP-AES results (one gold binding site in the X-ray structure and 11 AF molecules per hsFt chain suggested via ICP-AES data) demonstrated that a significant fraction of AF is not directly bound to the protein, but it is encapsulated in the bulk of the hsFt nanocage.

### 2.3. Cytotoxicity Studies

The anticancer efficacy of AF has been proven in different tumors, including several types of carcinomas [4,25,26]. In this work, the cytotoxicity of _AF_hHFt and _AF_hsFt was determined on a cancer cell line, the human epidermoid carcinoma (A431), and on a normal one, immortalized human keratinocytes (HaCaT). Cells were incubated with increasing concentrations of _AF_hHFt, _AF_hsFt and AF. After a 48 h incubation, cell viability was evaluated via the MTT assay [27] and the respective IC_50_ values, corresponding to the drug concentration able to reduce the cell viability by 50%, were determined (Table 2). By comparing the IC_50_ values obtained for the two gold-loaded nanocomposites with those obtained from treating cells with the free drug, it was possible to assess the effect of the protein on the toxicity and the selectivity of the metal compound. _AF_hHFt was the system endowed with lower selectivity, as its IC_50_ value on immortalized cells was much lower when compared to _AF_hsFt and even more so to AF. On the contrary, _AF_hsFt preserved almost the same cytotoxicity of AF on the cancer cell line, but its selectivity was also decreased when compared to free AF.

### 2.4. Oxidative Stress Analysis

**Figure 6:**
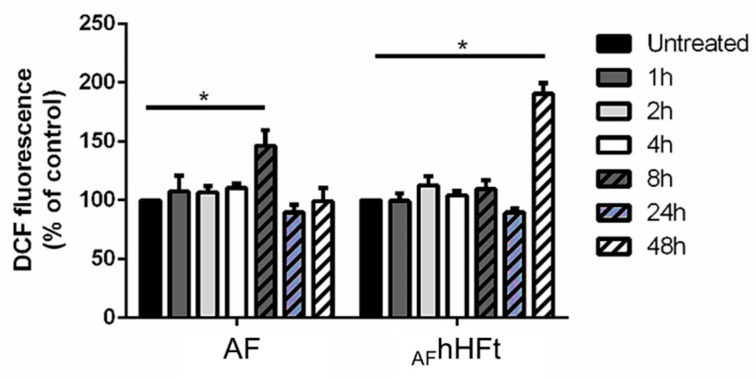
Time-course experiment to determine the effect of AF and _AF_hHFt on intracellular ROS levels of A431 cells. Cells were incubated in presence of both AF and _AF_hHFt for 1 h (dark grey bars), 2 h (light grey bars), 4 h (white bars), 8 h (dashed dark grey bars), 24 h (dashed blue bars), 48 h (dashed white bars). Black bars refer to the untreated cells. The fluorescence intensity of the probe is related to the intracellular ROS level and is reported as a percentage of untreated cells (%). Data shown are the means ± S.D. of three independent experiments and * indicates *p* < 0.05 with respect to untreated cells.

The effect of hsFt on the pro-oxidant activity of metallodrugs leading to apoptosis activation has been investigated in several studies [16,23,28], while what happens when a metallodrug is in part encapsulated within the hHFt nanocage and in part bound on the outer surface of hHFt is not known. For this reason, we studied the ability of AF and _AF_hHFt to alter the redox state of A431 cells by measuring the intracellular ROS levels and the variation in the mitochondrial membrane potential, which are two biological markers for oxidative stress. In this respect, it is useful to recall that the mechanism of action of AF includes its pro-oxidant activity, related to the activation of apoptosis [11]. As shown in Figure 6, AF was able to induce a significant increase in intracellular ROS levels after 8 h incubation, whereas _AF_hHFt was able to significantly increase intracellular ROS levels after a 48 h incubation. 

**Figure 7:**
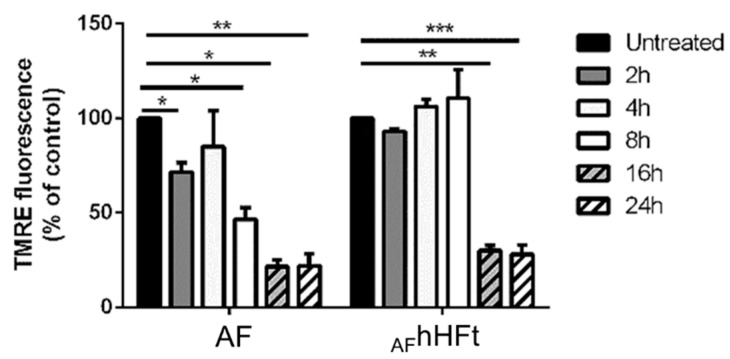
Changes in the mitochondrial membrane potential (Δψm) of A431 cells upon incubation with both AF and _AF_hHFt. Cells were incubated for 2 h (dark grey bars), 4 h (light grey bars), 8 h (white bars), 16 h (dashed dark grey bars), 24 h (dashed white bars). Black bars refer to the untreated cells. The fluorescence intensity of the probe related to Δψm is reported as a percentage of the control (%). Data shown are the means ± S.D. of three independent experiments and * indicates *p* < 0.05, ** indicates *p* < 0.005, *** indicates *p* < 0.001 with respect to untreated cells.

It is known that mitochondria are sensitive to changes in the cellular redox status and ROS activation is known to induce the depolarization of the mitochondrial membrane [29]. For this reason, the mitochondrial membrane potential of A431 cells upon AF and _AF_hHFt treatment was measured via a TMRE assay [30] over time (Figure 7). A431 cells were incubated for 2, 4, 8, 16 and 24 h with an amount of AF and _AF_hHFt corresponding to their IC_50_ values. In both cases, treatment resulted in the dissipation of the mitochondrial membrane potential (Δψm). However, for the free drug, a decrease in fluorescence intensity was observed already after 8 h incubation; whereas a strong depolarization started to occur after 16 h when cells were treated with _AF_hHFt. The lowest permeability threshold found after 16 h of treatment remained constant up to 24 h of incubation. This implies mitochondrial damage during treatment with AF and _AF_hHFt.

These results are in line with those obtained with the alteration in ROS levels, thus suggesting that _AF_hHFt acts more slowly than the free drug.

Preliminary experiments on the mechanism of the action of _AF_hHFt have also been carried out. Data suggest cell death induced by apoptosis, as observed in the case of cells that are treated with hHFt nanocages carrying high doses of doxorubicin [31], but further experiments are needed to verify these preliminary results.

## 3. Discussion

Gold-based compounds represent an alternative chemotherapeutic strategy to other well-known metal-based compounds, such as cisplatin or carboplatin, since they allow us to overcome several limitations associated with the platinum-based treatment [9]. Among gold complexes with cytotoxic activity, AF has attracted great interest. AF is an FDA-approved drug for the treatment of rheumatoid arthritis [1] that has shown potent anticancer activity against many cancer cells [2,3,4]. The efficacy of this molecule is related to its ability to alter the intracellular redox equilibrium, producing an increase in oxidative stress, which triggers the apoptosis process. Since it has been demonstrated that AF can interact with proteins [10,11], here we evaluated its encapsulation within two Ft nanocages [17,18,32,33]. The present study had three main aims: (1) to study whether AF encapsulation within a suitable nanocarrier could improve the performance of this already effective drug; (2) to obtain information about AF’s mode of interaction with proteins; and (3) to gain knowledge about possible gold binding sites on hHFt, whose structure with gold atoms has never been solved. In this respect, it should be recalled that there are only a few X-ray structures of adducts formed upon the reaction of AF with proteins [34,35,36,37] and only a few studies on metalated hHFt [24,38]. Indeed, hsFt has already been used to encapsulate several metallodrugs [20,21,23,24,28] and the cytotoxicity of the gold compound-encapsulated hsFt systems has already been studied [21,22,28], while works on anticancer gold compounds encapsulated with hHFt have never been published.

Thus, _AF_hHFt and _AF_hsFt have been produced and characterized; their cytotoxicity against a cancer and an immortalized cell line has been evaluated. The presence of Au within hHFt and hsFt was confirmed through ICP-AES analysis. hsFt can encapsulate about 270 AF molecules, while hHFt can encapsulate about 90 AF molecules. The encapsulation of AF within Fts does not significantly affect the overall structure of the proteins, as revealed through CD spectra and structural studies: they retain their secondary structure content and can reassemble in the typical spherical arrangement.

In both AF-loaded systems, the presence of the cage does not improve the performance of AF. The two nanocomposites exert a cytotoxicity that is comparable to or lower than that of the free AF, in line with what was observed in the case of other metallodrug-loaded ferritins [20,21,23,24,28]. However, a decrease in selectivity was also detected for both cages, with _AF_hHFt presenting the same cytotoxicity against normal and cancer cells. Another result of the paper is a first insight into the mechanism of action of _AF_hHFt. The study on the pro-oxidant activity of AF and _AF_hHFt in A431 cells suggests that the presence of the cage slows down the ability of AF to alter the redox state of the cells, but to a higher level with respect to the free drug. Both AF and _AF_hHFt induce apoptotic cell death. 

Although we do not know exactly how AF can be released by the nanocage, we expect that the action of _AF_hHFt could be associated with the release of the metal complex in the acidic endosome compartment, as suggested by us [16,23] and other authors [39] for other drugs encapsulated within Ft nanocages.

The identification of gold binding sites in the structures of the two AF-loaded nanocomposites confirms the high gold affinity for sulfur donors and the tendency of AF to act as gold (I) ions releasing prodrugs. In fact, in both _AF_hsFt and _AF_hHFt structures, the gold atoms are found close to the side chains of cysteine residues. This result is in agreement with what has been found in other structures of AF-protein adducts [34,35,36,37]. However, the two nanocomposites present significant differences in the distribution of gold binding sites, and this may provide a molecular basis to explain the results of the cytotoxicity experiments. In the structure of _AF_hsFt, a gold atom is bound to Cys126, buried at the interface between two hsFt subunits. The gold binding site close to Cys126 is not surprising since it has been already observed in the X-ray structures of other gold-encapsulated hsFts [21,28]. Considering that 270 molecules of AF are encapsulated within hsFt and just one gold binding site has been identified on the _AF_hsFt structure, it can be concluded that many loaded AF molecules are trapped within the bulk. This suggests that _AF_hsFt has a high number of AF molecules that can be released from its bulk and that it preserves the features of the native protein and thus its ability to be recognized by the Scara5 receptor (see Figure 5A) [18]. On the contrary, in _AF_hHFt, only a small fraction of AF molecules is within the bulk. Furthermore, in the structure of _AF_hHFt, the gold binding sites are mainly found on the outer surface of the cage (see Figure 4A), where Cys90 and Cys102 are located [40]. This result suggests that the features of the outer surface of hHFt (for example, the electrostatic potential) significantly change in the presence of AF. These findings could affect the hHFt’s capability to be recognized by TfR-1, thus explaining the loss of selectivity of _AF_hHFt.

## 4. Materials and Methods

### 4.1. Materials

AF and hsFt were purchased from Merck/Sigma-Aldrich and used without further purification. hHFt was expressed and purified as described by Ruzzenenti and coworkers with minor modifications [41].

### 4.2. Preparation and Spectroscopic Characterization of Auranofin-Encapsulated Ferritins

Encapsulation of AF inside the Ft cages was obtained by first disassembling both Fts into subunits at pH 13 by gently adding 2.0 M NaOH, and then reconstituting them at neutral pH by using 1.0 M sodium phosphate buffer pH 7.4 for _AF_hsFt and 1.0 M Tris HCl pH 7.4 for _AF_hHFt [20]. Specifically, protein samples of concentration 20 mg mL^−1^ dissociated at pH 13 were incubated in the presence of AF in a protein chain to metallodrug molar ratio of 1:20 for 1h under stirring. After incubation, the pH was raised to the physiological value to let the cages reassemble. After half an hour, samples were centrifuged for 10 min at 5000 rpm to remove possible precipitates, then ultracentrifuged at 10,000 rpm on Amicon Centricon centrifugal filters (cut-off 50 KDa), to remove the unbound drug, and washed with 10 mM sodium phosphate buffer pH 7.4 and 20 mM Tris HCl pH 7.4, respectively.

Fts concentrations were determined with the BCA protein assay (BCATM Protein Assay Kit, Pierce) [42].

UV-vis absorption spectra of _AF_hsFt and _AF_hHFt were recorded using a 0.1 cm optical path-length quartz cell on a JASCO V-560 UV-vis spectrophotometer in the range of 240–700 nm, using a protein concentration of 0.25 mg mL^−1^ in 10 mM sodium phosphate buffer pH 7.4 and 20 mM Tris HCl pH 7.4, respectively. Other experimental parameters were bandwidth 2.0 nm, scanning speed 200 nm min^−1^ and data pitch 1.0 nm.

Far-UV CD spectra were recorded on a Jasco J-715 spectropolarimeter equipped with a Peltier thermostatic cell holder (Model PTC-348WI) in the range of 190–250 nm, using protein concentration of 0.05–0.03 mg mL^−1^ in 10 mM sodium phosphate buffer pH 7.4 and 20 mM Tris HCl pH 7.4, and a 0.1 cm path length quartz cuvette. Each spectrum was obtained by averaging three scans and converting the signal to mean residue ellipticity in units of deg cm^2^ dmol^−1^. Other experimental settings were scanning speed 50 nm min^−1^, bandwidth 2.0 nm, resolution 1.0 nm, sensitivity 50 mdeg and response 2 s.

### 4.3. ICP-AES Measurements

The determination of metal concentration in the _AF_hsFt and _AF_hHFt nanocages was performed as previously reported [38] by using a Varian 720-ES inductively coupled plasma-atomic emission spectrometer equipped with a CETAC U5000 AT+ ultrasonic nebulizer, in order to increase the method sensitivity. An amount of 200 µL of each sample was used. The samples were transferred into polyethylene vials and digested in a thermo-reactor at 80 °C for 8 h with 2 mL of HNO_3_ 69.5% suprapure grade. Ultrapure water (≤18 MΩ) was added to the vials containing the _AF_hsFt and _AF_hHFt solutions until a final volume of 6 mL. All the samples were spiked with 1 ppm of Ge used as an internal standard and analyzed. Calibration standards were prepared by gravimetric serial dilution from a commercial standard solution of Au at 1000 mg L^−1^. The following wavelengths were used: 242.795 and 267.595 nm for Au and 209.426 nm for Ge. The operating conditions were optimized to obtain maximum signal intensity, and between each sample, a rinsed solution of 2.0 mL of HNO_3_ 69.5% suprapure grade and 4.0 mL of ultrapure water was used to avoid any “memory effect”.

### 4.4. Crystallization, X-ray Diffraction Data Collection, Structure Solution and Refinement

Crystals of _AF_hsFt were grown through the hanging drop vapor diffusion method using a reservoir solution of 0.6–0.8 M (NH_4_)_2_SO_4_, 0.1 M Tris HCl pH 7.4–7.7, 50–60 mM CdSO_4_ and protein concentration of 7.0 mg mL^−1^. Crystals of _AF_hHFt were grown using the same method and a reservoir solution containing 2.0 M MgCl_2_ and 0.1 M Bicine buffer pH 9.0, and a protein concentration of 19.0 mg mL^−1^.

Diffraction data were registered at 100 K at the XRD2 beamline of Elettra synchrotron, Trieste, Italy, using λ = 1.00 Å. Crystals were cryoprotected using a solution of the reservoir with 25% glycerol. Data were indexed, integrated and scaled using Autoproc [43]. Data collection statistics are reported in Table 1. The phase problem was solved by molecular replacement using the ferritin structures deposited in the PDB under the accession codes 5ERK [20] for hsFt and 5N27 [24] for hHFt as the starting models. Several rounds of restrained energy minimization and individual or mixed anisotropic/isotropic B-factor refinements were carried out using Refmac5 [44], Refinement cycles were followed by manual intervention based on observation of the electron density map carried out using Coot [45]. Refinement statistics are reported in Table 1. Au atom position was identified by analysis of anomalous difference electron density map. In this respect, it should be noted that since the weak sulfur anomalous f’ signal at 0.25 electrons is significantly visible in the anomalous difference electron density map (see, for example, SG atom of Cys130 in Figure 4C), one can confidently place gold occupancies down to ~10% (gold has an f’ signal of 9.5 electrons at 1.00 Å wavelength). Model geometry was validated using the PDB validation server. Figures with electron density maps were drawn with PyMOL (DeLano Scientific LLC, San Carlos, CA, USA). X-ray structures of _AF_hsFt and _AF_hHFt were deposited in the PDB [46] under the accession codes 8B7L and 8B7O, respectively.

### 4.5. Cytotoxicity Experiments

Immortalized human keratinocytes (HaCaT) were from Innoprot (Biscay, Spain) and human epidermoid carcinoma cells (A431 cells) were obtained from ATCC. Cells were cultured in Dulbecco’s modified Eagle’s medium (DMEM) (Sigma-Aldrich, St Louis, MO, USA), supplemented with 10% fetal bovine serum (HyClone), 2 mM L-glutamine and antibiotics (Sigma-Aldrich), under a 5% CO_2_ humidified atmosphere at 37 °C. For cytotoxic analyses, cells were seeded in 96-well plates at a density of 2.5 × 10^3^ cells per well. Then, 24 h after seeding, increasing concentrations of AF, _AF_hHFt and _AF_hsFt were added to the cells. After 48 h incubation, cell viability was assessed via the MTT (3-(4,5-dimethylthiazol-2-yl)-2,5-diphenyltetrazolium bromide) assay, as previously reported [47].

### 4.6. Oxidative Stress Analysis

To estimate ROS production, the protocol described by Petruk [48] was followed. Briefly, HaCaT and A431 cells were incubated with AF and _AF_hHFt for different lengths of time (1–48 h) and then incubated with 2′,7′-dichlorodihydrofluorescein diacetate (H_2_-DCFDA, Sigma-Aldrich). Fluorescence intensity was measured using a PerkinElmer LS50 spectrofluorometer (525 nm emission wavelength, 488 nm excitation wavelength, 300 nm min^−1^ scanning speed and 5 nm slit width for both excitation and emission). ROS production was expressed as a percentage of DCF fluorescence intensity of the sample under test, with respect to the untreated sample. Each value was assessed by three independent experiments, each with three determinations. Significance was determined by Student’s *t*-test.

The variation in the mitochondrial membrane potential (Δψm) was measured as described by Monti et al. [28]. Cells were plated at a density of 2 × 10^4^ cells per well and were treated after 2, 4, 8, 16 and 24 h, as described above. Subsequently, the cells were incubated with 200 nM cationic lipophilic dye tetramethylrhodamine ethyl ester (TMRE) for 20 min at 37 °C. Then, the cells were gently washed with 0.2% BSA in PBS three times and the fluorescence was measured in a microplate reader with peak Ex/Em = 549/575 nm. Each value is the mean of three independent experiments, with three measurements for each experiment. Significance was determined by Student’s t-test.

## 5. Conclusions

Auranofin was encapsulated for the first time within two distinct ferritin nanocages. The gold-loaded nanocomposites were analyzed from a biophysical, structural and biological point of view. The results showed that the 3D structures of the proteins are not affected by drug encapsulation. The analysis of the X-ray structures allowed us to identify the gold binding sites and to explain the biological activity of the two nanocomposites, whose performance is not improved when compared to that of the free drug.

## Figures and Tables

**Table 1:** Data collection and refinement statistics for AF-encapsulated Fts.

Data Collection Statistics *		
	**_AF_hHFt**	**_AF_hsFt**
PDB code	8B7O	8B7L
Space group	F432	F432
Unit cell parameters a = b = c (Å)	184.03	181.99
Molecules per asymmetric unit	1	1
Wavelength (Å)	1.00	1.00
Observed reflections	6523319 (300927)	5410903 (265838)
Unique reflections	90295 (4445)	73628 (3593)
Resolution (Å)	46.01–1.17 (1.19–1.17)	64.34–1.24 (1.26–1.24)
Completeness (%)	100.0 (100.0)	100.0 (100.0)
Rmerge	0.156 (3.112)	0.122 (3.122)
Rpim	0.018 (0.378)	0.014 (0.364)
Rmeas	0.157 (3.135)	0.123 (3.144)
I/σ(I)	26.6 (2.1)	34.9 (2.1)
Multiplicity	72.2 (67.7)	73.5 (74.0)
CC_1/2_	1.000 (0.777)	1.000 (0.772)
**Refinement Statistics**		
Resolution (Å)	46.01–1.17	64.34–1.24
N° reflections in working set	85177	84436
N° reflections in test set	4431	4449
N° non-H atoms in the refinement	2063	1964
R factor/Rfree (%)	0.150–0.166	0.160–0.180
B-factor overall (Å^2^)	13.74	15.89
Ramachandran Values (%)		
In favored regions	98.28 (114)	96.08 (98)
Outliers	0.00 (0)	0.00 (0)
R.m.s.d. from Ideality		
R.m.s.d. bonds (Å)	0.017	0.020
R.m.s.d. angles (°)	2.14	2.24

* Values in parenthesis refer to highest resolution shell.

**Table 2:** IC_50_ values expressed as gold concentration (μM) after 48 h of incubation with AF, _AF_hsFt and _AF_hHFt (based on the concentration of AF obtained via ICP-AES) on HaCaT and A431 cell lines.

	AF	_AF_hHFt	_AF_hsFt
HaCaT	10.2 ± 0.4	1.1 ± 0.2	4.8 ± 1.5
A431	1.0 ± 0.1	1.3 ± 0.2	1.7 ± 0.6

## Data Availability

Coordinates and structure factors for _AF_hsFt and _AF_hHFt were deposited in the PDB under the accession codes 8B7L and 8B7O, respectively.
